# Regional Variability of Lifestyle Factors and Hypertension with Prediabetes and Newly Diagnosed Type 2 Diabetes Mellitus: The Population-Based KORA-F4 and SHIP-TREND Studies in Germany

**DOI:** 10.1371/journal.pone.0156736

**Published:** 2016-06-08

**Authors:** Doris Stöckl, Ina-Maria Rückert-Eheberg, Margit Heier, Annette Peters, Sabine Schipf, Christine Krabbe, Henry Völzke, Teresa Tamayo, Wolfgang Rathmann, Christa Meisinger

**Affiliations:** 1 Institute of Epidemiology II, Helmholtz Zentrum München, German Research Center for Environmental Health, Neuherberg, Germany; 2 German Center for Diabetes Research (DZD e.V.), Partner Neuherberg, Neuherberg, Germany; 3 Competence Network Diabetes mellitus (Federal Ministry of Education and Research, Germany), Sites Düsseldorf, Munich and Greifswald, Germany; 4 Department of Obstetrics and Gynaecology, Klinikum Großhadern, Ludwig-Maximilians-University, Munich, Germany; 5 Institut für Allgemeinmedizin, Klinikum der Universität München, Ludwig-Maximilians-University, Munich, Germany; 6 Institute for Community Medicine, University Medicine Greifswald, Greifswald, Germany; 7 DZHK-German Centre for Cardiovascular Research, partner site Greifswald, Greifswald, Germany; 8 Institute of Biometrics and Epidemiology, German Diabetes Centre, Leibniz Center for Diabetes Research at Heinrich-Heine-University, Düsseldorf, Germany; 9 Central Hospital of Augsburg, MONICA/KORA Myocardial Infarction Registry, Augsburg, Germany; University of Utah School of Medicine, UNITED STATES

## Abstract

**Objective:**

Type 2 diabetes mellitus (T2D) shows regional differences in Germany. The purpose of the project was to compare the prevalence of prediabetes and undiagnosed T2D in two regions in Germany, the Northeast and the South, and to evaluate their associations with regional variations in lifestyle factors and hypertension.

**Methods:**

Data from the KORA-F4 study (South Germany, 2006–2008) and the SHIP-TREND study (Northeast Germany, 2008–2012) were used. Participants aged 35–79 years without T2D with an overnight fasting of more than 8 hours and an oral glucose tolerance test were included: KORA-F4: n = 2,616 and SHIP-TREND: n = 1,968.

**Results:**

The prevalence of prediabetes/newly diagnosed T2D was especially high in men (about 60%) and women (about 50%) in the Northeast, followed by men (about 50%) and women (about 30%) in the South. Lifestyle factors associated with T2D varied between the regions: more participants in the Northeast were active smokers and the percentages of people with overweight or obesity were greater than in their southern counterparts. However, these differences could not explain the striking disparity in prediabetes/newly diagnosed T2D. The frequency of hypertension was also distinctly higher in the Northeast than in the South and clearly associated with prediabetes/newly diagnosed T2D. Especially in men living in the Northeast, screening individuals with blood pressure ≥ 140/90mmHg might reveal up to 70% of those with prediabetes/newly diagnosed T2D.

**Conclusions:**

Knowledge about regional variability in T2D and related risk factors is important for the planning of diabetes prevention programs. In our analyses, common lifestyle factors did not nearly explain these variations between the northern SHIP-TREND and the southern KORA-F4 studies. Further examinations of regional socioeconomic, political, environmental and other aspects are needed. Meanwhile, targeted diabetes prevention strategies with a special focus on men living in the northern parts of Germany are reasonable.

## Introduction

In 2012 Schipf et al. reported regional variations of type 2 diabetes (T2D) prevalence in Germany, using population-based data of five regional studies. The numbers varied considerably between the different regions with the lowest prevalence of 5.8% in the South (KORA, Cooperative Health Research in the region of Augsburg study) and the highest prevalence with 12.0% in the East (CARLA, Cardiovascular Disease Living and Ageing study). The prevalence in the Northeast (SHIP, Study of Health in Pomerania) was 10.9%. [1]. The incidence rates, which were published subsequently, paralleled the prevalence differences [2]. Moreover, analogous differences were described for prediabetes and newly diagnosed T2D (NDD) in a recent analysis based on pooled population-based data from the same regions in the Northeast and South of Germany by Tamayo et al. [3]: Men and women in the Northeast of Germany had a considerably higher prevalence of prediabetes (in particular isolated impaired fasting glucose (i-IFG) and the combination of impaired fasting glucose (IFG) and impaired glucose tolerance (IGT)) and NDD than their counterparts in the South of Germany. In particular, prediabetes was more prevalent at a younger age in the Northeast. [3]. The authors implied that variance in lifestyle between the two studies did not explain this finding sufficiently and further analyses were needed. Furthermore, several attendant studies with these and other data showed for the first time that a factor other than personal attributes of the study participants, i.e. ‘regional deprivation’ was independently associated with T2D risk [4–6]. Knowledge about regional differences in prediabetes and related risk factors is necessary for the future planning of diabetes prevention programs and it is of interest to identify groups at high risk.

The aim of this analysis was to evaluate the influence of lifestyle factors such as smoking and physical activity, as well as hypertension, on regional variability in prediabetes and NDD in two regions in Germany (Northeast and South).

## Methods

### 1. Study populations

#### 1.1 The KORA-F4 study (South of Germany)

The KORA-F4 study (2006–2008) contains 7-year follow-up data of the KORA-S4 study (1999–2001), a population-based health survey conducted in the city of Augsburg and the two surrounding counties. For the S4 baseline study, a total sample of 6,640 subjects was drawn from the target population consisting of all German residents of the region aged 25 to 74 years. The study design, sampling method and data collection have been described in detail elsewhere [7, 8]. Of the 4,261 participants in S4, 3,080 took part in the F4 follow-up study resulting in a response of 79.6%. In the baseline KORA-S4 study, an oral glucose tolerance test (OGTT) was performed to all participants aged 55–74 without known T2D, but not to younger participants [9]. In the KORA-F4 study an OGTT was performed to all participants without previously diagnosed T2D and who did not take blood glucose lowering drugs [9, 10]. The investigations were carried out in accordance with the Declaration of Helsinki, including written informed consent of all study participants. The study was approved by the Ethics Committee of the Bavarian Medical Association.

#### 1.2 The SHIP-TREND study (Northeast of Germany)

Information for the SHIP-TREND study was collected between 2008 and 2012. A stratified random sample of 8,826 adults aged 20–79 years with German nationality was drawn from a central population registry of Western Pomerania. 4,420 individuals participated in the study, with a resulting response of 50.1%. All participants provided written informed consent and the medical ethics committee of the University of Greifswald approved the study protocol. The study design, sampling method and data collection of the SHIP-TREND study have been previously published [3, 11, 12].

### 2. Oral glucose tolerance test and classification of prediabetes and T2D

After an overnight fast of at least 8 h (only drinking water was allowed, participants should not smoke and avoid physical exercise before the examination), all non-diabetic participants underwent a standard 75g OGTT (Dextro OGT, Boehringer Mannheim, Mannheim, Germany). Blood was collected without stasis, refrigerated to 4–8°C and shipped on refrigerant packaging within 4 to a maximum of 6 h to the laboratory. Fasting glucose and 2 hour glucose were measured using a hexokinase method. In KORA-F4 serum and in SHIP-TREND plasma was examined. In KORA-F4, GLU flex by Dade Behring (Marburg, Germany) and in SHIP-TREND Dimension Vista by Siemens Healthcare Diagnostics (Eschborn, Germany) were used.

In order to determine whether both samples were comparable, duplicate measurements were carried out using serum samples of all SHIP-TREND participants. Both measurements (plasma and serum glucose) were highly correlated (r = 0.99; p<0.0001). In Passing Bablok regression analysis of serum versus plasma glucose (mmol/l) an intercept of 20.10 (95%CI 20.10; 20.10) and a slope of 1 were observed concluding that plasma values in SHIP-TREND might be only slightly lower than serum values. Furthermore, 30 serum blood glucose samples were drawn at random from KORA-F4 and reassessed in the SHIP-TREND laboratory, yielding a correlation coefficient of r = 0.94 (p<0.0001). On average, these original 30 KORA-F4 measurements were only slightly lower (mean -0.06 mmol/l; SD 0.17) when they were re-analyzed in the SHIP-TREND laboratory. Thus, serum glucose from KORA-F4 and plasma glucose from SHIP-TREND were considered as comparable for the current analysis [3].

Participants with fasting glucose values <5.6 mmol/l (<100 mg/dl) and 2 h glucose <7.8 mmol/l (<140 mg/dl) were defined as having normal glucose tolerance (NGT). Fasting glucose values ≥7.0 mmol/l (≥126 mg/dl) or 2 h glucose ≥11.1 mmol/l (≥200 mg/dl) were classified as NDD. Prediabetes was diagnosed if fasting glucose values ranged between 5.6 and 6.9 mmol/l (100–125 mg/dl, isolated impaired fasting glucose i-IFG) and/or 2 h postload glucose values between 7.8 and 11.0 mmol/l (140–199 mg/dl, isolated impaired glucose tolerance, i-IGT). Participants were categorized to three groups of prediabetes (glucose disturbances): i-IFG, i-IGT, and combined IFG and IGT (IFG/IGT) [13].

### 3. Data collection (interviews, anthropometry, laboratory measurements)

Information on socio-demographic variables, physical activity level, smoking habits, education and medication use was obtained by trained medical staff during a standardized face-to-face interview. Participants were classified as active during leisure time if they regularly participated in sports in the summer and winter and if they were active for > 1 h per week in both seasons. Participants were classified as non-smokers if they had either never smoked or less than one cigarette per day, as smokers if they smoked at least one cigarette per day regularly and as ex-smokers if they had stopped smoking at least 12 months ago.

Alcohol consumption was assessed differently in both studies—therefore, we did not include it. Educational level was defined by highest self-reported schooling degree achieved. Educational level was classified as a dichotomous variable contrasting low or middle (less than university qualification) with high level (university qualification). Low and middle education were combined because school types in the former German Democratic Republic were different to those in the Federal Republic of Germany. All study participants were asked to bring original packaging of their medications used during the last 7 days before the study examination. Pharmaceutical control numbers, names etc. were recorded and ATC (Anatomical Therapeutic Chemical Classification System) codes assigned accordingly. In addition, all study participants underwent a standardized medical examination. All measurement procedures have been described in detail elsewhere [7].

Anthropometric measurements were taken after the participants had removed their shoes, heavy clothing and belts. Body height was measured to the nearest 0.1 cm and weight to the nearest 0.1 kg. Body mass index (BMI) was calculated as weight [kg] divided by height^2^ [m^2^]. Waist circumference was measured at the level midway between the lower rib margin and the iliac crest.

Hypertension was defined using the mean of the second and third blood pressure readings with SBP ≥ 140 mmHg and/or DBP ≥ 90 mmHg, or intake of anti-hypertensive medication in participants with a physician’s diagnosis of hypertension (“awareness”). Participants with hypertension were categorized into one of the following four subgroups: (1) aware (with physician’ diagnosis) and controlled treated to target levels of < 140/90 mmHg, (2) aware and treated, but not reaching target blood pressure values of < 140/90 mmHg, i.e. uncontrolled treated, (3) aware, but not treated, (4) unaware of hypertension. Thus, “awareness” of hypertension applied to participants in categories 1, 2 and 3, “treatment” applied to those in categories 1 and 2 and “control” to those in category 1. Actual hypertension was defined as blood pressure values ≥ 140/90 mmHg or the use of antihypertensive medication, given that the participants were aware of being hypertensive.

HDL-, LDL- and total cholesterol, and triglycerides were measured as described elsewhere [12, 14].

### 4. Study population

All participants aged 35 to 79 years were included in the study (KORA-F4: n = 2,929, SHIP-TREND: n = 3,491). Participants were excluded from the analyses if one of the following criteria was met: self-reported known T2D, or T2D treatment (KORA-F4: n = 225, SHIP-TREND: n = 411), missing information on known T2D (KORA-F4: n = 86, SHIP-TREND: n = 12), or missing or implausible values in glucose measurements (KORA-F4: n = 1, SHIP-TREND: n = 469). All participants who underwent glucose challenge testing were required to be in the fasting state (≥ 8 hours overnight fasting). In SHIP-TREND, however, 658 participants without known T2D had not been fasting for at least 8 hours. For the estimation of unknown T2D or prediabetes, participants who did not meet fasting criteria were excluded from the analysis. These “non-fasting” 658 participants from SHIP-TREND had a lower proportion of women (43.8% vs. 54.5%, p < 0.0001), higher mean levels of diastolic blood pressure (78.4 mmHg vs. 77.5 mmHg, p = 0.01), lower HDL-cholesterol (1.4 mmol/l vs. 1.5 mmol/l, p = 0.003), lower triglycerides (median 1.2 vs. 1.3 mmol/l, p < 0.0001) than the 1,942 participants with completed 8 h overnight fast. However, both groups from SHIP-TREND—fasting versus non-fasting participants—did not differ in age, BMI, total cholesterol, LDL-cholesterol, systolic blood pressure, fasting plasma glucose and 2 h-glucose, as previously described by Tamayo et al. [3]. Further information on lifestyle factors was missing for 13 participants (KORA-F4: n = 1, SHIP-TREND: n = 12). Therefore the analyses on prediabetes and NDD presented here are based on 2,616 participants from KORA-F4 (1,379 women; 1,237 men) and 1,968 participants from SHIP-TREND (1,073 women; 895 men).

### 5. Statistical analyses

Basic characteristics of the study population were analyzed. For continuous variables the means and standard deviations were calculated. For categorical variables numbers and percentages were reported. Logistic regression models were carried out to determine factors associated with prediabetes or NDD. Models were fitted adjusting for sex, age, education, BMI, physical activity, smoking, and hypertension. Interactions were tested by an interaction term.

Significance tests were two-tailed and p-values less than 0.05 were considered statistically significant. All analyses were performed using SAS (version 9.3, SAS Institute Inc, Cary, NC, USA).

## Results

### 1. Biometrical and lifestyle variables stratified by study, sex and diabetes status

In Table 1 the participant’s characteristics are shown stratified for study and sex: on average, people were about 55 years old and somewhat younger in SHIP-TREND than in KORA-F4, the mean BMI was slightly, but significantly higher in SHIP-TREND with 27.5 kg/m^2^ in women (27.0 kg/m^2^ in KORA-F4 women) and 28.7 kg/m^2^ in men (27.8 kg/m^2^ in KORA-F4 men). Likewise, both systolic and diastolic blood pressures were significantly elevated in SHIP-TREND compared to KORA-F4 (e.g. SBP in men: 133.2 mmHg vs. 127.2 mmHg). Fasting glucose and 2-hour glucose as measured during the OGTT examination were also higher on average in SHIP-TREND (e.g. 2-hour glucose in women: 6.7mmol/l vs. 6.1mmol/l). Lifestyle factors did not differ as clearly as the biometric measurements reported above: physical inactivity was somewhat more common among KORA-F4 women than SHIP-TREND women, but did not differ between men. The two studies had approximately the same percentage of ex-smokers within sex strata (while there were distinctly more ex-smokers among men than women). However, current smoking was reported significantly more often in SHIP-TREND than in KORA-F4 in both men and women with about 20%. Middle and low education was more common among KORA-F4 women than SHIP-TREND women, but did not differ among men.

**Table 1:** Description of the study populations separated by sex.

	Women			Men		
	KORA-F4	SHIP-TREND	p-value	KORA-F4	SHIP-TREND	p-value
N	1379	1073		1237	895	
age (years)	55.2 (12.2)	53.7 (11.2)	**0.002**	55.8 (12.4)	54.5 (11.6)	0.061
BMI (kg/m^2^)	27.0 (5.0)	27.5 (5.3)	**0.013**	27.8 (4.1)	28.7 (4.1)	**< 0.0001**
systolic blood pressure (mmHg)	116.5 (17.7)	121.1 (16.7)	**< 0.0001**	127.2 (17.2)	133.2 (15.5)	**< 0.0001**
diastolic blood pressure (mmHg)	73.1 (9.2)	75.0 (9.2)	**< 0.0001**	77.9 (10.1)	80.5 (9.7)	**< 0.0001**
fasting glucose (mmol/l)	5.1 (0.6)	5.4 (0.7)	**< 0.0001**	5.4 (0.6)	5.8 (1.0)	**< 0.0001**
2-hour glucose (mmol/l)	6.1 (2.0)	6.7 (2.1)	**< 0.0001**	6.3 (2.1)	6.8 (2.7)	**< 0.0001**
no physical activity ‘	29.5	25.7	**0.038**	31.5	32.2	0.720
current smoking°	16.5	20.8	**0.0043**	20.5	22.8	**0.038**
ex-smoking °°	30.2	30.0	0.4168	45.3	47.6	0.052
middle/low education	80.5	76.0	**0.0078**	70.8	68.7	0.3135

Data are expressed as mean ± Standard deviation or percentages.

P-values were calculated using T-tests for metric and univariable logistic regression analyses for categorical variables.

The sample was restricted to participants without known T2D and to participants who had been fasting for a minimum of 8 hours.

Abbreviations:

KORA-F4: Cooperative Health Research in the Region of Augsburg (2006–2008);

SHIP-TREND: Study of Health in Pomerania (2008–2012);

BMI: Body Mass Index

‘no physical activity: Participants were classified as non-active if they participated in sports for < 1 hour per week in summer and winter.

° current smoking: Participants smoked at least one cigarette per day.

°° ex-smoking: Participants had quit smoking at least 12 months ago.

Table 2 reports characteristics of the participants by OGTT-status group (NGT, i-IFG, i-IGT, IFG-IGT, NDD) again stratified by sex and study. Women and men in SHIP-TREND were almost twice as often affected by prediabetes or NDD than their counterparts in the KORA-F4 study. With 60%, the prevalence was especially high in SHIP-TREND men, SHIP-TREND women and KORA-F4 men had similar frequencies (about 50%), while KORA-F4 women were characterized by the lowest percentage (about 30%). Interestingly, more SHIP-TREND than KORA-F4 participants were indeed affected with i-IFG, IFG-IGT and NDD, however, they did less often suffer from i-IGT.

**Table 2:** Characteristics by NGT, i-IFG, i-IGT, IFG-IGT, NDD, stratified by sex.

	NGT	i-IFG	i-IGT	IFG-IGT	NDD	Total
**Total N (%)**						
*KORA Women*	996 (72.2)	155 (11.2)	104 (7.5)	77 (5.6)	47 (3.4)	1,379 (100)
*SHIP Women*	630 (58.7)	211 (19.7)	74 (6.9)	97 (9.0)	61 (5.7)	1,073 (100)
*KORA Men*	694 (56.1)	303 (24.5)	69 (5.6)	108 (8.7)	63 (5.1)	1,237 (100)
*SHIP Men*	356 (39.8)	309 (34.5)	35 (3.9)	116 (13.0)	79 (8.8)	895 (100)
**Age (years) mean (std)**						
*KORA Women*	52.4 (11.6)	61.4 (10.4)	62.5 (11.8)	64.4 (8.2)	63.9 (9.8)	55.2 (12.2)
*SHIP Women*	50.9 (10.8)	56.7 (9.6)	53.0 (11.6)	60.5 (10.6)	62.5 (8.3)	53.7 (11.2)
*KORA Men*	52.4 (12.1)	57.7 (11.4)	62.4 (11.8)	63.3 (9.8)	64.5 (9.8)	55.8 (12.3)
*SHIP Men*	51.8 (11.8)	53.7 (10.7)	57.9 (12.4)	59.8 (11.0)	60.7 (10.0)	54.5 (11.6)
**BMI (kg/m**^**2**^**)mean (std)**						
*KORA Women*	26.0 (4.6)	28.7 (5.4)	29.1 (5.2)	30.5 (4.6)	31.6 (4.0)	27.0 (5.0)
*SHIP Women*	26.2 (4.5)	29.0 (5.7)	28.3 (4.4)	30.6 (5.6)	30.8 (6.2)	27.5 (5.3)
*KORA Men*	26.7 (3.6)	28.4 (3.8)	28.9 (4.5)	30.4 (5.0)	30.9 (4.6)	27.8 (4.1)
*SHIP Men*	27.1 (3.6)	29.1 (3.8)	29.9 (3.2)	30.1 (4.0)	31.7 (4.4)	28.7 (4.1)
**BMI >30 kg/m**^**2**^ **% (n)**						
*KORA Women*	17.3 (172)	32.9 (51)	38.5 (40)	54.6 (42)	66.0 (31)	24.4 (336)
*SHIP Women*	18.8 (118)	38.4 (81)	37.8 (28)	47.4 (46)	47.5 (29)	28.2 (302)
*KORA Men*	14.7 (102)	31.4 (95)	37.7 (26)	44.4 (48)	53.2 (33)	24.6 (304)
*SHIP Men*	17.1 (61)	34.0 (105)	42.9 (15)	44.8 (52)	59.5 (47)	31.3 (280)
**Systolic blood pressure (mmHg) mean (std)**						
*KORA Women*	113.6 (16.6)	123.8 (18.3)	121.3 (21.1)	125.8 (15.5)	127.7 (13.6)	116.5 (17.7)
*SHIP Women*	117.1 (14.9)	124.8 (16.5)	125.3 (16.3)	129.7 (18.9)	130.5 (19.8)	121.1 (16.7)
*KORA Men*	123.5 (15.8)	130.3 (17.6)	132.8 (20.6)	133.4 (16.2)	135.5 (17.8)	127.2 (17.2)
*SHIP Men*	129.5 (14.5)	132.8 (15.1)	140.5 (11.6)	139.2 (15.2)	139.1 (17.9)	133.2 (15.5)
**Diastolic blood pressure (mmHg) mean (std)**						
*KORA Women*	72.5 (9.2)	75.1 (8.6)	73.4 (9.3)	75.6 (8.8)	74.5 (9.2)	73.1 (9.2)
*SHIP Women*	73.8 (8.7)	76.5 (9.0)	78.3 (10.0)	76.6 (10.2)	75.8 (10.4)	75.0 (9.2)
*KORA Men*	76.9 (9.5)	79.0 (10.4)	79.1 (12.0)	79.9 (10.5)	79.8 (11.2)	77.9 (10.1)
*SHIP Men*	79.0 (10.0)	80.9 (9.1)	84.6 (8.6)	82.2 (9.2)	81.6 (10.5)	80.5 (9.7)
**Blood pressure > = 140/90 mmHg, % (n)**						
*KORA Women*	9.6 (95)	16.1 (25)	17.3 (18)	14.3 (11)	21.3 (10)	11.6 (159)
*SHIP Women*	10.0 (63)	19.1 (40)	24.3 (18)	26.3 (25)	31.2 (19)	15.5 (165)
*KORA Men*	15.0 (104)	31.4 (95)	36.2 (25)	35.2 (38)	44.4 (28)	23.5 (290)
*SHIP Men*	24.7 (88)	33.9 (104)	65.7 (23)	49.6 (57)	43.0 (34)	34.3 (306)
**Physically non-active % (n)** ‘						
*KORA Women*	27.1 (270)	34.2 (53)	32.7 (34)	40.3 (31)	40.4 (19)	29.5 (407)
*SHIP Women*	25.1 (158)	24.6 (52)	31.1 (23)	28.9 (28)	24.6 (15)	25.7 (276)
*KORA Men*	29.9 (201)	29.4 (89)	46.4 (32)	37.0 (40)	42.9 (27)	31.5 (389)
*SHIP Men*	27.5 (98)	34.6 (107)	45.7 (16)	30.2 (35)	40.5 (32)	32.2 (288)
**Current smoking % (n)** °						
*KORA Women*	18.7 (186)	13.6 (21)	7.7 (8)	9.1 (7)	10.6 (5)	16.5 (227)
*SHIP Women*	24.6 (155)	22.8 (48)	8.1 (6)	7.2 (7)	11.5 (7)	20.8 (223)
*KORA Men*	25.4 (176)	15.8 (48)	15.9 (11)	5.6 (6)	19.1 (12)	20.5 (253)
*SHIP Men*	23.9 (85)	25.6 (79)	11.4 (4)	19.8 (23)	16.5 (13)	22.8 (204)
**Ex smoking % (n)** °°						
*KORA Women*	31.0 (309)	32.9 (51)	26.9 (28)	19.5 (15)	27.7 (13)	30.2 (416)
*SHIP Women*	30.8 (194)	32.2 (68)	31.1 (23)	21.7 (21)	26.2 (16)	30.0 (322)
*KORA Men*	38.6 (268)	52.2 (158)	52.2 (36)	56.5 (61)	58.7 (37)	45.3 (560)
*SHIP Men*	43.5 (155)	46.6 (144)	54.3 (19)	56.0 (65)	54.4(43)	47.6 (426)
**Low/middle education % (n)**						
*KORA Women*	77.9 (775)	84.5 (131)	89.3 (92)	87.0 (67)	91.5 (43)	80.5 (1108)
*SHIP Women*	74.7 (470)	77.3 (163)	75.3 (55)	83.5 (81)	73.8 (45)	76.0 (814)
*KORA Men*	67.0 (463)	74.6 (226)	78.3 (54)	72.(78)	82.5 (52)	70.8 (873)
*SHIP Men*	66.6 (237)	71.2 (220)	62.9 (22)	67.2 (78)	73.4 (58)	68.7 (615)

Abbreviations:

KORA-F4: Cooperative Health Research in the Region of Augsburg (2006–2008),

SHIP-TREND: Study of Health in Pomerania (2008–2012),

BMI: Body Mass Index,

NGT: normal glucose tolerance,

i-IFG: impaired fasting glucose,

i-IGT: impaired glucose tolerance,

NDD: newly diagnosed diabetes,

‘ no physical activity: Participants were classified as non-active if they participated in sports for < 1 hour per week in both season.

° current smoking: Participants smoked at least one cigarette per day.

°° ex-smoking: Participants had quit smoking at least 12 months ago.

People with prediabetes or NDD tended to be approximately ten years older than their non-diabetic counterparts. Mean BMI was distinctly higher in those with prediabetes/NDD in all stratification groups—thus, the percentage of those with a BMI ≥ 30 kg/m^2^ was also much greater in prediabetes and highest in NDD compared to the normo-glycaemic group. The same was true for systolic and diastolic blood pressure measurements which, furthermore, increased consistently across stratification groups: KORA-F4 women had the lowest blood pressure readings, followed by SHIP-TREND women and KORA-F4 men—SHIP-TREND men had the highest values in each diabetes status group respectively. Accordingly, the percentages of people with uncontrolled hypertension (BP ≥ 140/90 mmHg) were smallest in KORA-F4 women with normal OGTT (9.6%) and highest in SHIP-TREND men with i-IGT (65.7%). No physical activity (or less than 1h/week) was more common among those with prediabetes/NDD than NGT controls. Current smoking showed a reverse pattern with the highest numbers of smokers in the NGT group; ex-smoking had no clear association in women, while in men the percentage of ex-smokers was higher in those with prediabetes/NDD than in those with NGT. Low or middle educational status (as compared to high education) tended to be more frequent in people with prediabetes/NDD, especially in women and men from the KORA-F4 study.

### 2. Logistic regression analyses

#### 2.1 Factors associated with prediabetes/NDD

In a univariate logistic regression model (Table 3), SHIP-TREND participants had an OR = 1.82 (95% CI 1.61–2.05) for having prediabetes or NDD compared to KORA-F4 participants. When the model was adjusted for age and sex, the odds even increased to 2.19 (95%CI 1.93–2.50), since SHIP-TREND participants were more often female and somewhat younger than KORA-F4 participants. Likewise in this model, men had an OR = 2.16 (95%CI 1.90–2.46) for prediabetes/NDD compared to women and age was also significantly related (OR = 1.06, 95%CI 1.05–1.07 per year) to the prediabetes/NDD risk. In the full model, SHIP-TREND participants still had an OR = 2.03 (95% CI 1.77–2.33), while the covariates sex, age, BMI, hypertension and physical activity were also significantly related. Educational status and smoking turned out non-significant, but were kept in the analyses for completeness.

**Table 3:** Logistic regression analyses.

Variable	OR (95% CI) for prediabetes/NDD (no T2D as reference)	p-value
**A: Univariable model:**		
SHIP-TREND (vs. KORA-F4)	**1.82 (1.61–2.05)**	<.0001
**B: Adjusted for sex and age:**		
SHIP-TREND (vs. KORA-F4)	**2.19 (1.93–2.50)**	<.0001
men (vs. women)	**2.16 (1.90–2.46)**	<.0001
age (continuous)	**1.06 (1.05–1.07)**	<.0001
**C: Fully adjusted model:**		
SHIP-TREND (vs. KORA-F4)	**2.03 (1.77–2.33)**	<.0001
men (vs. women)	**1.93 (1.68–2.23)**	<.0001
age (per year, continuous)	**1.05 (1.04–1.06)**	<.0001
Low + middle education (vs. high education)	1.08 (0.92–1.26)	0.3709
BMI 25–30 kg/m^2^ (vs. < 25 kg/m^2^)	**1.87 (1.58–2.22)**	<.0001
BMI > 30 kg/m^2^ (vs. < 25 kg/m^2^)	**4.31 (3.58–5.20)**	<.0001
BP ≥ 140/90mmHg (vs. < 140/90 mmHg)	**1.66 (1.40–1.95)**	<.0001
ex-smoker (vs. never-smoker)	0.98 (0.85–1.14)	0.8231
smoker (vs. never-smoker)	0.91 (0.75–1.10)	0.3375
< 1h physical activity (vs. > 1h)	**1.16 (1.01–1.35)**	0.0419
**c = 0.768**		

Physical activity in separate fully adjusted models (including non-significant variables education and smoking):

KORA-F4: OR = 1.03 (0.84–1.25, p = 0.8092)

SHIP-TREND: OR = 1.31 (1.05–1.63, p = 0.0180)

Physical activity in separate fully adjusted models (without non-significant variables):

KORA-F4: OR = 1.01 (0.83–1.23, p = 0.9455)

SHIP-TREND: OR = 1.33 (1.07–1.65, p = 0.0116)

A: Univariable model featuring the association of diabetes status and study,

B: adjusted for sex and age,

C: fully adjusted model, non-significant lifestyle variables were kept for completeness

#### 2.2 Interaction terms and a possible impact of physical activity

Interaction terms of one co-variable with study were all non-significant, except for age and physical activity, which was borderline significant in the fully adjusted model. In two logistic models, one for each study separately and adjusted for all other variables, the association of physical activity with prediabetes/NDD was non-significant in KORA-F4 (OR = 1.03, 95% CI 0.84–1.25), but significant in SHIP-TREND with an OR = 1.31 (1.05–1.63).

#### 2.3 Logistic regression analyses to identify differences between KORA-F4 and SHIP-TREND

Moreover, we performed logistic regression analyses with “study” as the main outcome (Table 4) to identify differences between KORA-F4 and SHIP-TREND that might in part explain the striking variance in the prevalence of prediabetes/NDD. In an unadjusted model, the OR of prediabetes/NDD vs. no T2D was 1.82 (95% CI 1.61–2.05) for SHIP-TREND with KORA-F4 as reference (same as in the univariate model above). Adding sex and age as covariables to a second model, the OR of prediabetes/NDD increased to 2.19 (95% CI 1.92–2.49) for SHIP-TREND. In the fully adjusted model including diabetes status, sex, age, hypertension, smoking, education, physical activity, and BMI the OR of prediabetes/NDD was still 2.03 (95%CI 1.77–2.32) for SHIP-TREND.

**Table 4:** Logistic regression analyses.

Variable	OR (95% CI) for SHIP-TREND (KORA-F4 as reference)	p-value
**A: Univariable model:**		
prediabetes/NDD (vs. no T2D)	**1.82 (1.61–2.05)**	<.0001
**B: Adjusted for sex and age:**		<.0001
prediabetes/NDD (vs. no T2D)	**2.19 (1.92–2.49)**	<.0001
men (vs. women)	0.82 (0.73–0.93)	0.0015
age (continuous)	0.98 (0.98–0.99)	<.0001
**C: Fully adjusted model:**		
prediabetes/NDD (vs. no T2D)	**2.03 (1.77–2.32)**	<.0001
men (vs. women)	**0.73 (0.64–0.83)**	<.0001
age (continuous)	**0.98 (0.97–0.99)**	<.0001
low+middle education (vs. high education)	**0.78 (0.68–0.90)**	0.0007
BMI 25–30 kg/m^2^ (vs. < 25 kg/m^2^)	**1.25 (1.08–1.46)**	0.0033
BMI > 30 kg/m^2^ (vs. < 25 kg/m^2^)	**1.28 (1.08–1.52)**	0.0052
BP ≥ 140/90 mmHg (vs. < 140/90 mmHg)	**1.50 (1.28–1.75)**	<.0001
ex-smoker (vs. never-smoker)	1.13 (0.99–1.30)	0.0800
smoker (vs. never-smoker)	**1.37 (1.16–1.62)**	0.0003
< 1h physical activity (vs. > 1h)	**0.86 (0.76–0.99)**	0.0325
**c = 0.623**		

A: Univariable model featuring the association of diabetes status and study,

B: adjusted for sex and age,

C: differences in diabetes status, lifestyle factors, and hypertension control between studies in the fully adjusted model

### 3. High risk cluster comparison

In order to assess differences in the clustering of risk factors between the two studies, we defined three high-risk cluster variations (Table 5). In SHIP-TREND women and men the percentage of people with prediabetes/NDD within the risk cluster groups was always greater than the percentage of KORA-F4 women and men in the same groups. Physical activity reduced the percentage of people with prediabetes/NDD in SHIP-TREND high risk clusters, especially in women, but not in KORA-F4 women and men.

**Table 5:** High-risk cluster comparison between KORA-F4 and SHIP-TREND.

	Women (% prediabetes or NDD (n))		Men (% prediabetes or NDD (n))	
	KORA-F4	SHIP-TREND	KORA-F4	SHIP-TREND
BMI < 25 kg/m^2^ + BP < 140/90 mmHg	12.9 (66)	22.8 (81)	22.4 (61)	28.7 (37)
BMI < 25 kg/m^2^ + BP ≥ 140/90 mmHg	26.3 (10)	36.7 (11)	53.9 (21)	42.9 (9)
BMI 25–30 kg/m^2^ + BP < 140/90 mmHg	28.3 (121)	38.7 (120)	36.2 (171)	53.7 (167)
BMI 25–30 kg/m^2^+ BP ≥ 140/90 mmHg	35.5 (22)	63.0 (46)	59.6 (87)	69.3 (106)
BMI ≥30 kg/m^2^+ BP < 140/90 mmHg	47.7 (132)	57.6 (136)	62.3 (124)	78.1 (103)
BMI ≥30 kg/m^2^ + BP ≥ 140/90 mmHg	54.2 (32)	72.6 (45)	74.3 (78)	78.0 (114)
Smoker + BMI > 25 kg/m^2^ +BP ≥ 140/90 mmHg	46.2 (6)	56.3 (9)	60.7 (17)	76.1 (35)
Ex-smoker + BMI > 25 kg/m^2^ + BP ≥ 140/90 mmHg	36.6 (15)	75.5 (37)	69.2 (99)	73.1 (114)
Non-smoker, irrespective of BMI and BP	27.3 (362)	39.4 (397)	40.1 (426)	56.3 (390)
Physically inactive + BMI > 25 kg/m^2^ + BP ≥ 140/90 mmHg	45.1 (23)	74.3 (26)	65.6 (61)	75.3 (67)
Physically active + BMI > 25 kg/m^2^ + BP ≥ 140/90 mmHg	44.3 (31)	65.0 (65)	65.8 (104)	72.3 (141)
all other combinations	26.2 (329)	37.6 (352)	38.4 (377)	54.2 (331)

### 4. Hypertension frequency, awareness, treatment, and control

Hypertension treatment and control varied considerably between sexes and studies. Table 6 shows all possible hypertension groups, stratified accordingly: 70% of the KORA-F4 women were not affected with hypertension of which 20% had prediabetes/NDD. In SHIP-TREND women, 59% did not have hypertension and 28% of those had prediabetes/NDD. For men, the numbers were 59% without hypertension (31% of these with prediabetes/NDD) in KORA-F4 and 45% (47% of these with prediabetes/NDD) in SHIP-TREND respectively. Controlled hypertension was more frequent in SHIP-TREND than in KORA-F4 (e.g. in women; 26% vs. 18%), however, the percentage of people with prediabetes/NDD within this group was also higher in SHIP-TREND. Uncontrolled hypertension was most frequent in SHIP-TREND men (14%), known but untreated hypertension was reported most often in KORA-F4 men (8%). Finally, unknown hypertension occurred twice as often in SHIP-TREND men than in KORA-F4 men (13 vs. 6%) and about twice as often in SHIP-TREND women as in KORA-F4 women (5% vs. 3%). In all subgroups the percentage of people with prediabetes/NDD was greater in SHIP-TREND than in KORA-F4. Thus, the highest risk of prediabetes/NDD was found in SHIP-TREND men with uncontrolled hypertension (79%).

**Table 6:** Hypertension treatment status stratified by sex and study.

	Women		Men	
	KORA-F4	SHIP-TREND	KORA-F4	SHIP-TREND
**no hypertension (%)**	70.1 (19.9)	58.6 (28.2)	59.3 (31.2)	44.6 (47.1)
**controlled (%)**	18.4 (49.4)	26.0 (58.1)	17.5 (60.8)	21.1 (69.2)
**Uncontrolled (%)**	4.3 (50.9)	8.3 (63.6)	8.8 (77.6)	13.8 (78.9)
**known, not treated (%)**	4.6 (34.9)	2.6 (50.0)	8.4 (58.3)	7.6 (63.2)
**unknown hypertension (%)**	2.6 (31.4)	4.6 (65.3)	6.1 (54.1)	12.9 (67.8)

The percentage of participants with prediabetes or NDD within each treatment group is included in brackets.

In Table 7 all percentages were calculated referring to participants with hypertension instead of all participants in order to allow for or simplify comparisons with other studies.

**Table 7:** Hypertension treatment status in study participants with hypertension (known or unknown) stratified by sex and study.

	Women		Men	
	KORA-F4	SHIP-TREND	KORA-F4	SHIP-TREND
**N with hypertension (% of all participants)**	408 (29.9)	442 (41.4)	498 (40.7)	494 (55.4)
**controlled (%)**	61.5 (49.4)	62.7 (58.1)	43.0 (60.8)	38.1 (69.2)
**Uncontrolled (%)**	14.5 (50.9)	19.9 (63.6)	21.5 (77.6)	24.9 (78.9)
**known, not treated (%)**	15.4 (34.9)	6.3 (50.0)	20.7 (58.3)	13.8 (63.2)
**unknown hypertension (%)**	8.6 (31.4)	11.1 (65.3)	14.9 (54.1)	23.3 (67.8)

The percentage of participants with prediabetes or NDD within each treatment group is indicated in brackets.

**Fig 1:**
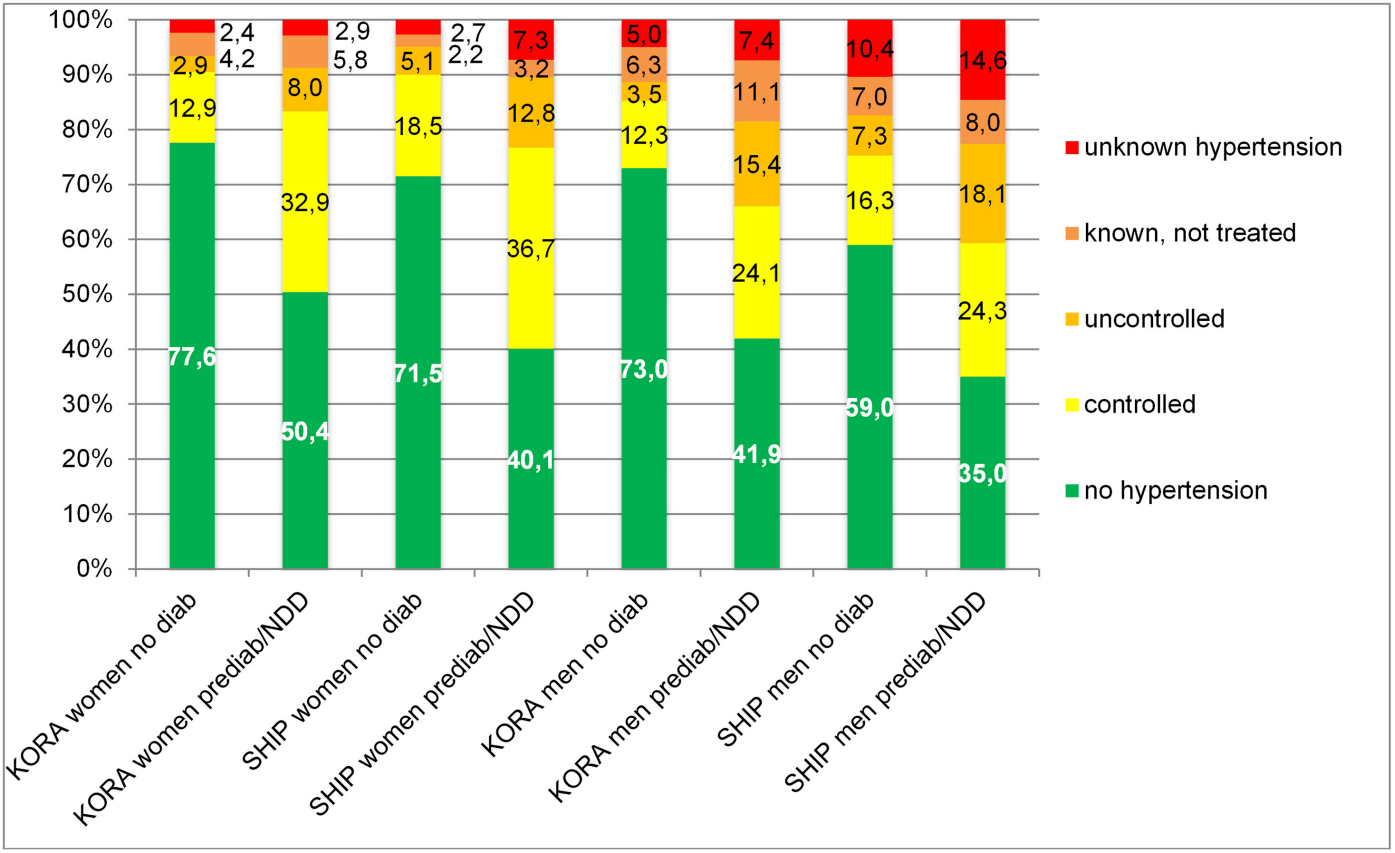
Hypertension and hypertension treatment status stratified by study, sex and diabetes status. NDD: newly diagnosed T2D.

In Fig 1 the association is illustrated the other way round featuring simultaneous stratification of study, sex and diabetes status and the percentages of hypertension treatment groups within these strata. Hence, the proportion of people not affected by hypertension was consistently smaller in groups with prediabetes/NDD, women in KORA-F4 and SHIP-TREND were more often controlled treated than men, while the proportion of people with unknown hypertension was rather small in women and greatest in SHIP-TREND men with prediabetes/NDD with 15%.

**Fig 2:**
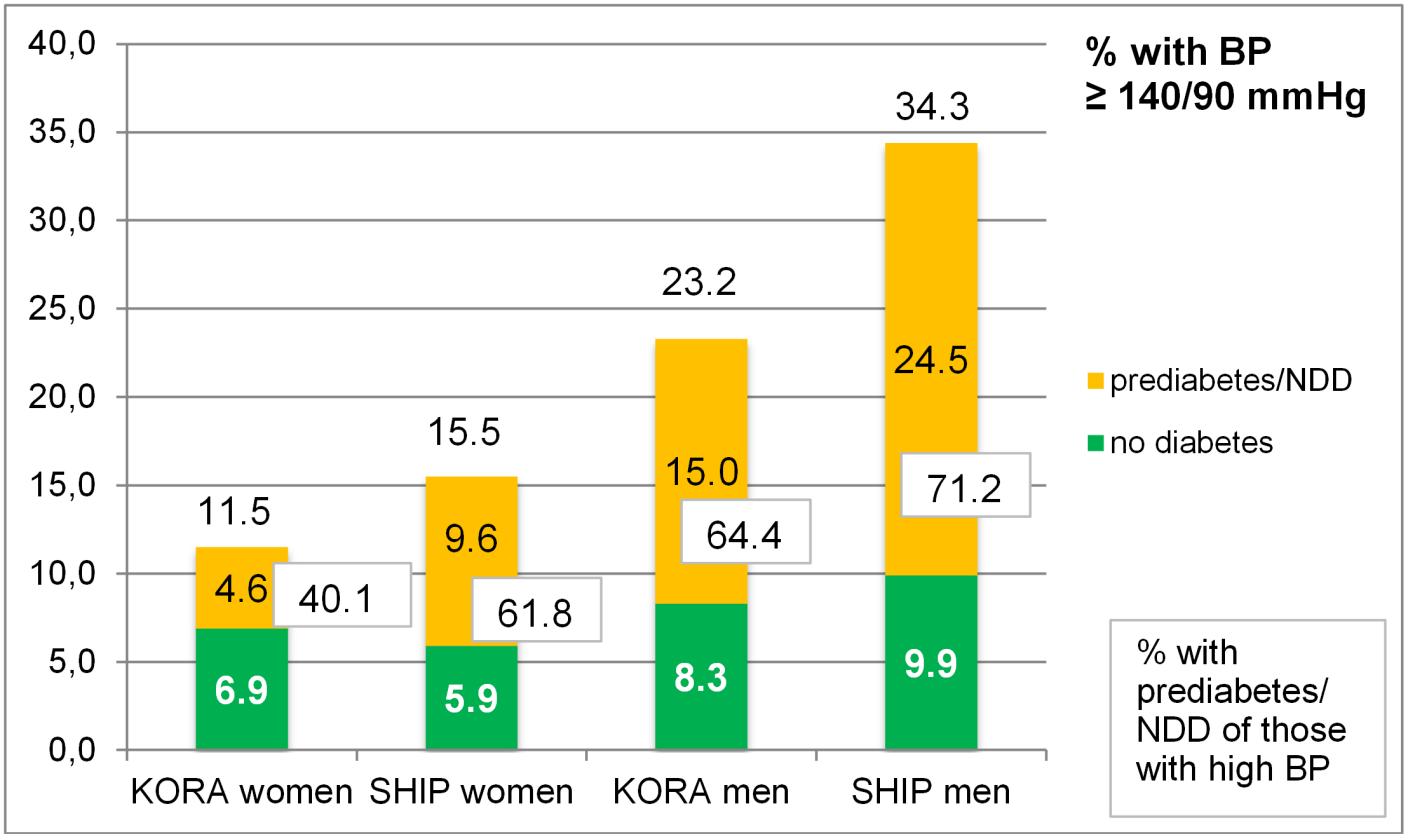
Participants (%) with uncontrolled hypertension stratified by study and sex. Within each stratum the percentage of those with prediabetes/NDD is included (numbers in boxes). BP: blood pressure, NDD: newly diagnosed T2D.

Fig 2 shows the percentages of participants with study blood pressure measurements ≥ 140/90 mmHg, split by diabetes status: 11.5% of KORA-F4 women had current hypertension—4.6% with prediabetes/NDD and 6.9% without prediabetes/NDD—thus, 40% of those with current hypertension had prediabetes/NDD. Of all SHIP-TREND women, 15.5% had current hypertension (5.9% without T2D and 9.6% with prediabetes/NDD), thus 61.8% of those with hypertension had prediabetes/NDD. In men analogous numbers were clearly higher with 23.2% of KORA-F4 men having hypertension (64.4% of these had prediabetes/NDD) and 34.3% of SHIP-TREND men with hypertension (71.2% of these had prediabetes/NDD).

## Discussion

### 1. Key results

The main results of our analyses conclude as follows: The prevalence of prediabetes/NDD was about two-fold higher in the Northeast than in the South. BMI and hypertension went closely hand in hand with prediabetes/NDD, however, these two variables and all lifestyle factors included in the models were not able to explain the striking disparity in prediabetes/NDD frequencies between both regions. Physical activity might pose a protective factor in the Northeast, but was not significantly associated in the southern population. Hypertension frequency is considerably higher in the Northeast than in the South, awareness is better in the South, while both regions are quite similar concerning treatment and control. However, especially in men, there is much room for improvement.

### 2. BMI, physical activity, and smoking

Normal weight, physical activity, and non-smoking are known to be inversely associated with prediabetes/NDD and T2D, and improving one’s lifestyle accordingly can prevent progression to overt T2D or even reverse pre-diabetic states to normal glucose metabolism (e.g.[15]).

On the occasion of the 20^th^ and 25^th^ anniversaries of the German reunification, comprehensive reports by the Robert-Koch-Institute (RKI) in Berlin reviewed and evaluated differences in health status, risk factors, health behavior, health care parameters etc. between federal states of the former German Democratic Republic and of the Federal Republic of Germany [16, 17]. According to these publications, people in the German Democratic Republic, which includes the SHIP-TREND study region, were characterized by a higher frequency of adverse lifestyle factors such as overweight and obesity, physical inactivity, alcohol consumption and smoking (especially in men) and were disadvantaged in terms of health care and health outcomes.

Thus, life expectancy was higher in the West than the East in 1991/1993, but has almost equalized since then in women, while there is still a difference of more than one year in men [17]. The mean BMI was higher in the East around 1990, but has increased all over Germany in the recent decades, which is partly due to the aging of the population, leading to a convergence of Eastern and Western regions in Germany [17, 18]. However, Mecklenburg-Western Pomerania (including the SHIP-TREND study region) has had the top ranking position of all German federal states concerning overweight in 2005 with 57.4% (as compared to 55.6% in Saxony-Anhalt, 53.8% in Thuringia, 41.2% in Hamburg, 44.5% in Berlin) [19, 20].

Engagement in physical activity was less frequent in the German Democratic Republic shortly after reunification, however, it has increased in both the West and the East of Germany and the difference has leveled out as reported by the German Health Interview and Examination Survey for Adults (DEGS) [17, 21].

Interestingly, ex-smoking poses a greater risk for T2D than active smoking which has been assumed to be due to compensatory intake of high-caloric food and a consequent increase in body weight (Stein, 2014 [22]). However, even irrespective of weight gain after quitting, an increased risk was reported in Japanese women and men (Oba, 2012 [23]). In the beginning of the 1990s, women in the East smoked considerably less than women in the West, while men smoked more frequently in the East than the West at that time [17]. Smoking was also more frequent in SHIP-TREND than in KORA-F4, however, in our analyses, the association of smoking or ex-smoking with prediabetes/NDD was not significant.

Alcohol consumption was not analyzed in the recent study because of different definitions in KORA-F4 and SHIP-TREND, but according to national surveillance by the Robert Koch—Institute the frequency of high consumption was higher in the East than the West of Germany before and for some time after reunification [16, 17].

An analysis featuring the comparison of two independent surveys of the SHIP study, SHIP-0 (1997–2001) and SHIP-TREND (2008–2012), also found that smoking and alcohol consumption declined over time, while BMI and the proportion of participants with obesity increased [24].

### 3. Blood pressure control

Hypertension is a major issue in health care all around the world, because of its rising prevalence and its tight interconnection with overweight, T2D and cardiovascular diseases (most importantly myocardial infarction and stroke), which today are the most prominent causes of death. Its role as a risk factor for incident T2D has been established [25]. In a previous analysis with data from the precursor studies to the studies used in our analyses, KORA-S4 (1999–2001) and SHIP-0 (1997–2001), Meisinger et al. already found a much higher prevalence of hypertension in the Northeast of Germany (SHIP-0, men: 60.1%, women: 38.5%) compared to the South (KORA-S4, men: 41.4%, women: 28.6%). This difference has persisted in the follow-up study KORA-F4 and the new study SHIP-TREND. However, awareness, treatment and control were very similar in both comparisons, indicating that the quality of health care was not the reason for the prevalence differences. Lifestyle factors associated with hypertension were not discussed then.

Interestingly, Germany in particular has a very high prevalence of hypertension compared to other European countries and the United States of America—a finding that has not yet been appreciated and analysed sufficiently [26]. In recent years, improvements of blood pressure control in Germany have been documented—however, the management of this grave disease can still be optimized [27].

### 4. Further possible risk factors for prediabetes

In SHIP-TREND women and men the percentage of people with prediabetes/NDD within high risk cluster groups was always greater than the percentage of KORA-F4 women and men in the same groups. This observation indicates that hitherto unknown or disregarded risk factors operated in the Northeast or that the participants in the South did benefit from protective factors we did not look at.

A body of literature is available on risk factors for and on the prevalence of prediabetes and T2D in different countries and ethnicities, however, not much is known about regional variability within otherwise largely homogeneous populations. Thus, risk factors other than individual characteristics and lifestyle issues have hardly been discussed. In a previous publication, our study group already collected continuative ideas about risk factors on a societal level that might have influenced the differential T2D prevalence in northern and southern Germany. Since our study participants were born during the years of division of Germany into the German Democratic Republic and the Federal Republic of Germany, a variety of differences due to the very different political and societal systems are conceivable: amongst others different diets [28] and variations of fetal and infant growth could have played a role [29]. Moreover, regional deprivation (and the unemployment rate in particular) has been shown to increase T2D prevalence independently from other known risk factors [4–6]. Though the East and the West of Germany have converged appreciably after the reunification in terms of health and economics, today, the East still has a higher unemployment rate, lower financial capacity, and lower life expectancy [30]. Moreover, in recent decades, the South of Germany has been known to be financially and demographically stronger than the North—an observation expressed by the newer phrase ‘South-North-gradient’ [17, 31]. Thus, persisting regional variability is probably due to persisting socioeconomic differences and future studies should concentrate on richer and poorer regions of Germany irrespective of East or West. A concrete example is the difference in the density of outpatient care units: in 2011 most general practitioners per 100,000 residents were registered in Bavaria, Hamburg and Berlin, while numbers were below national mean in Brandenburg and North-Rhine Westphalia. Moreover, within federal states, rural areas were disadvantaged compared to urban regions [32]. Interestingly, differences in historically grown traditions and mentalities have also been mentioned, which might influence the wellbeing of individuals in different geographical areas: e.g. in the North heteronomy and dependence of farmworkers, who were not even allowed to choose their fiancé, were predominant, while people in the South used to cultivate some own land independently and find own life strategies. This might have led to greater self-reliance, initiative and motivation in the South. In contrast, people in the North seem to give in to poverty more fatalistically [31].

Finally, westernized disease has been described to be sensitive to social deprivation, i.e. a person’s position in a social hierarchy. According to this, leaders experience a lower level of stress and less anxiety due to a greater sense of control, which protects them from stress-borne metabolic and cardiovascular disturbance [33]. This effect might also be found on the greater scale of geographical regions, national states and political systems.

### 5. Strengths and limitations

The cross-sectional design of the study represents a limitation, implicating that cause and effect relationships cannot be discerned. Furthermore, although we adjusted for a variety of important confounding variables, we cannot exclude that unknown risk factors may have biased the present analysis. Residual confounding cannot entirely be excluded. KORA-F4 is the follow-up study to KORA-S4, and was conducted about seven years later. At baseline, OGTT had been administered to participants aged 55 years and older, and identified people with newly diagnosed T2D at that time who were excluded from the recent analyses together with people with known T2D. Therefore, the previous publication of our study group separated the results additionally by age and nevertheless found much higher prevalence of prediabetes and NDD in SHIP-TREND compared to KORA-F4. In conclusion, these two studies are slightly different, from different time periods and prone to selection/recruitment bias as mentioned (50 vs over 70% response rates) which limits firm conclusions to be made on cross referential data. Finally, the reproducibility of the OGTT is not 100%, indicating that a certain number of people would be diagnosed differently after repeated testing on a different appointment day [34].

The strength of the study is the large number of subjects randomly drawn from the general population, and the availability of data on lifestyle and multiple risk factors, which were measured according to a standardized protocol.

### 6. Conclusion and Implication

Lifestyles in Eastern and Western regions of Germany have been equalizing in recent years, but not always turning to good account as in overweight and obesity. The past disadvantage in Eastern Germany still projects into the present and new South-North inequalities superimpose those circumstances. Further studies of potential explanatory factors for the disparities in the regional prevalence of prediabetes and T2D should focus on individual (e.g. blood pressure, BMI, lifestyle) and also on regional characteristics (e.g. pollution and noise, green spaces and traffic, extend of social inequality, unemployment rate, nearby recreational facilities such as swimming baths and playgrounds, number of general practitioners and specialists etc.). Our study indicates that the prevention of T2D must not only focus on individual people but on the environments and social systems people live in. In other words not only prevention in terms of behavior oriented prevention, but structural prevention is needed. Finding ways to shape the world in view of social equality and a healthy environment, which supports a lifestyle that matches the requirements of human physiology and psychology while allowing for maximal individual freedom, poses a great challenge for now and the future.
